# Supplementary material for: Dissociation of tone merger and congenital amusia in Hong Kong Cantonese
Source: PLoS One. 2021 Jul 1;16(7):e0253982. doi: 10.1371/journal.pone.0253982 (PMC8248700; doi:10.1371/journal.pone.0253982)
Supplement: Attachment — Submitted filename: Response to Reviewers.docx [file pone.0253982.s002.docx]

# Response to Reviewers

Reviewer #1:

This paper presents a large and carefully-conducted study on tone merger in Hong Kong Cantonese. In general I recommend publication, but have some comments about the interpretation, and discussion of the related literature.
***Response*:** Thank you very much for reviewing our manuscript and for the constructive feedback, which has helped us to strengthen the manuscript. We have carefully revised the interpretation and discussion of the related literature according to the specific suggestions below. Hope these revisions have satisfactorily addressed all the concerns.

p. 27 lines 9 – 16. The claim that speakers of Mandarin are less sensitive to tone height is at variance with the findings of Deutsch Henthorn and Dolson, 2004 (Music Perception 2004, 21, 339-356) that speakers of Mandarin showed remarkable pitch consistency in reciting the same list of twelve words on two different days. Out of 15 subjects, five showed averaged pitch differences of less the 0.25 semitone, and another 5 showed averaged pitch differences of 0.25-0.50 semitone. As in Cantonese, both pitch height and contour are determinants of tone in Mandarin.

***Response*:** Thank you for pointing out this missing citation, which is highly relevant to the issue of speech-music relationship. We have now added this reference in the discussion. While Mandarin speakers showed great F0 consistency in lexical tone *production* according to the above mentioned study, please note that several cross-linguistic studies have demonstrated that Mandarin speakers are *perceptually* more sensitive to pitch contour cues than pitch height cues (Chandrasekaran, Gandour, & Krishnan, 2007; J. Gandour, 1983; J. T. Gandour & Harshman, 1978). Furthermore, when asked to perceive (non-native) Cantonese contour-level and level-level tonal contrasts, English listeners were found to outperform Mandarin listeners in using pitch height to discriminate the level-level tone pairs (Qin & Jongman, 2016). Last, as there are level as well as contour tones in the Cantonese tone system, an event-related potentials (ERPs) study on Cantonese revealed delayed mismatch negativity (MMN) and reduced MMN amplitude toward a native tone pair with a pitch contour contrast (T1-T2, high level-high rising tone) compared to another native tone pair with a pitch height contrast (T6-T1, low level-high level tone) in Cantonese listeners (Tsang, Jia, Huang, & Chen, 2011). Altogether these studies support the notion of greater sensitivity of Cantonese listeners to pitch height differences. To make these points clear, we have made the following changes in the discussion (p. 27 line 24 to p. 28 line 11):

“As a result, Cantonese speakers are presumably more *perceptually* sensitive to small pitch height differences, whereas Mandarin speakers demonstrated better *perceptual* sensitivity to pitch contour differences (in terms of tone production Mandarin speakers have been found to demonstrate sensitivity to F0 height, showing F0 consistency in tone production (Deutsch et al., 2004)). This explanation is also in line with the findings of several cross-linguistic studies on tone perception that Cantonese listeners placed more weight on pitch height cues, in contrast to Mandarin listeners who placed more weight on pitch direction cues (J. Gandour, 1983; J. T. Gandour & Harshman, 1978; Z. Qin & Jongman, 2016). Furthermore, an event-related potentials (ERPs) study revealed delayed mismatch negativity (MMN) and reduced MMN amplitude toward a native tone pair with a pitch contour contrast (T1-T2, high level-high rising tone) compared to another tone pair with a pitch height contrast (T6-T1, low level-high level tone) in Cantonese listeners (Tsang, Jia, Huang, & Chen, 2011), which also supports the notion of greater sensitivity of Cantonese listeners to pitch height differences.”

p.30. lines 2-3, and line 11. The invocation of attention-switching requires explanation. In what way does attention-switching explain the findings? An increase in tone merging among young adult speakers of Cantonese would appear to be a good explanation of the findings. Since several different tone languages are spoken in Hong Kong, one would expect to find an impact of exposure to these different languages. The authors might want to examine this in a further study, in which they investigate the linguistic backgrounds of the subjects in detail.

***Response*:** Thanks for these suggestions. Regarding the role of attention switching in tone merging, Ou and colleagues argued that sluggish attentional switch may prevent the automatic attentional system from disengaging itself fast enough from one sound to the next, leading to degraded sensory analysis and auditory representation in tone merging individuals (Ou, Law, & Fung, 2015; Ou & Law, 2017). This explanation has now been added in the revised manuscript (p. 32 line 24 to p. 33 line 1).

Regarding the influence of other tone languages, we have added the following discussion accordingly (p. 33, lines 16-22): “These variations may be partly driven by the highly diverse and multilingual environment in Hong Kong (Mok & Zuo, 2012), and to some extent the lack of a romanization system of spoken Cantonese in Hong Kong, which has been associated with lower phonological awareness, including tone awareness, in native language processing (Cheung, Chen, Lai, Wong, & Hills, 2001; Ho & Bryant, 1997; Shu, Peng, & McBride-Chang, 2008). Future studies may investigate how these factors interact with individual differences (e.g., in attentional switching) in driving the tonal variations.” It is an interesting idea to examine the linguistic backgrounds of the subjects, and in our mind a large-scale study should be conducted to examine the impact of various sociolinguistic factors including linguistic backgrounds on tone merger in Hong Kong Cantonese.

Reviewer #2:

This study investigated the relationship between music and speech by comparing Cantonese amusic participants with Cantonese participants who were merging tones in perception and/or production in a series of tasks testing their musical and lexical tone abilities. They would like to address whether amusia and tone merge were based on domain-general mechanisms or whether they were domain-specific. They found that amusic and merging participants had different patterns which support the domain-specific view. They concluded that their findings corroborate an earlier study which they have discussed in detail in the Introduction to motivate their study.
The study provides novel data which help explore the relationship between music and speech from a less-understood perspective of whether merging participants would have poorer musical abilities. The manuscript is clearly written and easy to follow. There are, however, some issues, mainly methodological, that need to be addressed in the revision, although stronger justification for their study is also needed.

***Response*:** Thank you very much for reviewing our manuscript and for the constructive feedback, which has helped us to strengthen the manuscript. We have carefully revised the manuscript according to the specific comments below, including addressing the methodological issues and providing more motivation for our study. Hope these revisions have satisfactorily addressed all the concerns.

p.6: ‘First, the merging and non-merging participants were selected based on the confusion of tones in production, and it is not clear whether they showed any perceptual confusion of tones.’ The authors can add ‘given that there are different types of merging speakers as discussed above’.
***Response*:** Added. Thanks.

p.6: ‘Thirdly and most importantly, it has not been investigated before whether merging individuals, especially those who confuse tones perceptually, would show inferior musical abilities and lower sensitivities to fine-grained pitch height and contour differences compared to non-merging individuals.’
This seems to be an important motivation of the study, but there is no clear exposition why this would be expected, if previous studies have shown the separation between music and speech. In addition to Mok & Zuo (2012) who reported merging speakers with advanced musical training, there are other similar studies which the authors did not discuss. For example: Maggu, Wong, Antoniou, Bones, Liu & Wong (2018) Effects of combination of linguistic and musical pitch experience on subcortical pitch encoding. Journal of Neurolinguistics, 47: 145, and the relevant studies reviewed in there. The authors need to elaborate more on why they would have that hypothesis in the first place, even though they have more discussion later about amusia which is not the same issue.
***Response*:** This is an excellent point. It is indeed important to further justify our hypothesis that tone merging individuals would show *inferior* musical abilities and domain-general pitch sensitivities compared to non-merging individuals. To this end, the following discussion has been added in the introduction (p. 6, lines 1-24):

“Previous studies have revealed a mixed picture when it comes to the question of whether tonal language speaker with musical training would have an additive advantage in their native speech perception. In contrast to the consistent findings of musical training or higher musical aptitude associated with better non-native tone perception or learning in *non-tonal language speakers* (C.-Y. Lee et al., 2014; Li & Dekeyser, 2017; Wong & Perrachione, 2007; Wong et al., 2007; Zhao & Kuhl, 2015), results on the effect of musical training in *tonal language speakers* are mixed and inconclusive. While some study has revealed an additive effect of musical abilities and tonal language experience, reporting better native tone perception in Cantonese speakers with absolute pitch than those without (Maggu, Lau, Waye, & Wong, 2021), other studies have found no evidence for such additive effects (Maggu et al., 2018), or that the musician advantage is restricted to more trivial aspects such as the discrimination of within-category pitch distinctions (Wu et al., 2015; Zhu, Chen, & Yang, 2021) or processing speed (Tang et al., 2016). It is likely that the strong influence of top-down language knowledge in tonal language speakers has confined the effect of musical aptitude in native tone perception. The top-down influence may also explain the lack of musician advantage in tone-merging individuals in Mok & Zuo (2012) to some extent. In contrast to the mixed picture, it remains an open question whether tone-merging individuals, especially those confusing tones perceptually, would have degraded musical abilities and/or domain-general pitch sensitivity. Although top-down language knowledge might offer some protection, the influence of inferior musical abilities and/or domain-general pitch sensitivity is likely to be detectable. Indeed, tone confusion has been reported in Cantonese speakers with a musical disorder (see the next subsection on congenital amusia), which provides justification for this hypothesis. Without examining musical perception and pitch sensitivity in tone-merging individuals, it would not be possible to conclude confidently that tone merger is domain specific with little influence from the musical domain or domain-general pitch processing ability.”

p.10-11: ‘a group with poor perception and good production of T4-T6 or NM, a group with good perception and poor production of T2-T5 or PM, and a group with poor perception and poor production of T2-T5 or FM’ Why did they mix the two merging tone pairs here? Why did they not control for it? Please explain. Also, how about the other merging pair of T3/T6? They did not mention anything about this pair in the study. Why did they exclude it?
***Response*:** Thanks for these suggestions. It is indeed important to justify our selection criteria. We did not choose the T3-T6 pair because the merging of this pair primarily occurs in production but not in perception ([+per][-pro]). Central to our hypothesis, we wanted to focus on tone pairs that participated (at least) in perceptual merger. For this reason, we selected the T2-T5 pair, which showed various confusion patterns in perception and production ([-per][-pro] and [+per][-pro]). It would have been ideal to choose another group of individuals who showed confusion in perception but not production of T2-T5 ([-per][+pro]). However, individuals who showed this kind of confusion are rarely found in our pool of subjects as well as in previous reports (Bauer, Cheung, & Cheung, 2003; Law, Fung, & Kung, 2013; K. Y. S. Lee, Chan, Lam, Van Hasselt, & Tong, 2015; Yu, 2007). Therefore we chose another tone pair T4-T6 that showed confusion in perception but not production ([-per][+pro]). These justifications have now been added in the revised manuscript (p. 10, footnote 1).

p.13: ‘The participants’ tone discrimination accuracy was measured from the AX discrimination task.’ Please give more detail about this AX discrimination task.
***Response*:** The AX discrimination task for screening tone-merging individuals is the same as the tone discrimination task mentioned later. To avoid any misunderstanding, we have added the following: “These two tasks [tone production and AX discrimination task] were also used to assess tone production and discrimination abilities in amusical participants and to compare tone-merging individuals with amusics.” (p. 12, lines 14-6)

p.13: Please explain clearly why 80% was chosen as the threshold for dividing participants into different groups. 80% is well above the chance level (1/6 = 16.67% for all six tones, 1/3 = 33.33% for confusion between the two target tones). It is obviously that those with accuracy lower than 80% had poorer performance than control participants, but whether they should be classified as merging participants need more justification. Why did the authors not use their perception and production accuracy rates as continuous variables? More explanation is needed, as it is important in interpreting their results.
***Response*:** Thanks for these suggestions. The 80% cut-off was used because we wanted to capture more variances within each tone-merging group. As the tone merger in Hong Kong Cantonese is still on going and incomplete in the community, we have observed in our previous dataset a wide range of individual variances in the behavioral patterns, with the majority of subjects falling in between the two ends, i.e., ideal performance of 100% accuracy and chance-level performance. Although an 80% cut-off may seem high on the surface, we argue that deviation from the 'ideal' performance would already suggest some extent of confusion and reduced distinction in one's own phonology. For typically-developed adults, one would implicitly assume that they are capable of distinguishing their native phonemes with minimal errors, and expect a close to 100% accuracy for a simple AX task adopted here (which is indeed the pattern we observed in the control group). Moreover, the results of the tone discrimination and production tasks confirmed significant differences between tone-merging groups and controls. The aforementioned points have now been added in the revised manuscript (p. 13, lines 9-17).

We agree with the reviewer that the term “merger” implies a chance level confusion pattern. Thus we have replaced group labels like ‘full merger’, ‘near merger’ and ‘partial merger’ with descriptions of tone confusion in perception or production (e.g., [+per][-pro], [-per][+pro], and [-per][-pro]) throughout the manuscript. Although admittedly the labels [-pro] or [-per] might still imply chance-level performance to some readers, please note that we defined these labels as indicating poor tone production or perception, not chance-level performance (see p. 4, lines 6-9; p. 12, footnote 2).

‘Participants who had an accuracy below 80% in T2-T5 discrimination but more than 80% accuracy in T2-T5 production were classified into the PM group (T2-T5 [+per][-pro])’ Shouldn’t this group be classified as [-per][+pro]??? Have I missed something here?
***Response*:** Corrected. Thanks. The sentence has been rewritten as the following: “Participants who had an accuracy above 80% in T2-T5 discrimination but below 80% in T2-T5 production were classified into the [+per][-pro] group.” (p. 13, lines 3-5)

‘those who achieved lower than 80% accuracies in both T2-T5 perception and T2-T5 production were assigned into the FM group (T2-T5 [-per][-pro])’ Classifying someone who can distinguish T2 and T5 well above chance level as the FULL MERGER group is baffling. The same also applies to the PM and NM classifications. I think the authors should not simply adopt the terms used in Fung & Lee (2019) whose claims about the tone pairs being collapsed were too exaggerated.
***Response*:** Thanks for this suggestion. Please see our response above.

p.13: ‘Note that tone perception or production confusion is not part of the diagnostic criteria of amusia.’ So were any of their amusic participants merging tones or were any of the merging participants amusic? Please clarify.
***Response*:** This is exactly the question we tried to examine in this study. Here we were simply emphasizing that we did not prescreen amusic participants based on their tone perception or production performance (or tone merging participants based on their musical abilities). Otherwise the whole study would be circular.

p.14-15: The authors can include a figure to illustrate the various patterns in their experiment for easy reference.
***Response*:** Thanks for this great suggestion. We have created a figure (Figure 1) to help the readers better understand the pitch threshold task.

p.16: Was this AX tone discrimination task the same as the screening AX task mentioned on p.13??? If not, what’s the difference between the two tasks? Please clarify.
***Response*:** Yes it’s the same task. Please see our response above.

p.18: ‘We did not anticipate the confusion of T4 and T6 in production in any group, even for the NM group whose tone confusion concerns discrimination not production.’ Please clarify why.
***Response*:** We have clarified the point in the following sentences: “Recall that the three tone-merging groups in the current study included [+per][-pro] (T2-T5), [-per][+pro] (T4-T6), and [-per][-pro] (T2-T5). In other words, the only tone pair undergoing tone merging in *production* is T2-T5. For the T4-T6 pair, the [-per][+pro] (T4-T6) group only exhibited perceptual confusion of this pair but not its production. Therefore we did not anticipate the confusion of T4 and T6 in production in any tone-merging group.” (p. 18, lines 8-13)

p.18: ‘For T4 and T6, we analysed the F0 height (mean F0 across ten time points)’ The difference between T4 and T6 is mainly manifested in the second half of the tone. Why did the authors not include a more dynamic measure (e.g. slope) to compare these two tones but relied on overall F0 height? Please explain.
Also, the second half of T4 is often creaky. How was it treated in the acoustic measurement? This has a bearing on their tone space measurement (Tone space was obtained by subtracting the lowest F0 of T4 from the highest F0 of T1 in each participant) as the lowest F0 of T4 can vary between participants depending on how the creaky parts were treated.
***Response*:** Thanks for these suggestions. F0 measurements affected by creaky voice were disregarded from the analysis. This was mentioned on p. 18 line 52 – p. 19 line 1. Following the suggestion above, we also examined the F0 slope of T4 and T6. One-way ANOVA conducted on the T4-T6 slope difference (T4 minus T6) again revealed no significant group difference (*F*(4, 60) = .630, *p* = .643). The result of T4-T6 slope analysis has been added in a footnote (p. 23, footnote 3).

p.29: ‘One possible explanation is that the amusic participants recruited in the current study have rather severe impairments in musical pitch perception (see Figure 2).’ Reference is needed to support that the amusic participants in this study had severe impairments, or with more severe impairment that those in Shao et al., 2016 and Zhang & Shao, 2018.
***Response*:** References added. Thanks for this suggestion.

p.29: ‘A second pattern that is worth noting is that’ They should start a new paragraph here.

***Response*:** Done. Thanks for this suggestion.

**References**

Bauer, R. S., Cheung, K., & Cheung, P. (2003). Variation and merger of the rising tones in Hong Kong Cantonese. *Language Variation and Change*, *15*(02), 211–225. https://doi.org/10.1017/S0954394503152039

Chandrasekaran, B., Gandour, J. T., & Krishnan, A. (2007). Neuroplasticity in the processing of pitch dimensions: A multidimensional scaling analysis of the mismatch negativity. *Restorative Neurology and Neuroscience*, *25*(0), 195–210. Retrieved from http://www.ncbi.nlm.nih.gov/pmc/articles/PMC4380289/

Cheung, H., Chen, H. C., Lai, C. Y., Wong, O. C., & Hills, M. (2001). The development of phonological awareness: Effects of spoken language experience and orthography. *Cognition*, *81*(3), 227–241. https://doi.org/10.1016/S0010-0277(01)00136-6

Deutsch, D., Henthorn, T., & Dolson, M. (2004). Absolute Pitch, Speech, and Tone Language: Some Experiments and a Proposed Framework. *Music Perception*, *21*(3), 339–356. https://doi.org/10.1525/mp.2004.21.3.339

Gandour, J. (1983). Tone perception in Far Eastern languages. *Journal of Phonetics*, *11*(2), 149–175. https://doi.org/10.1016/s0095-4470(19)30813-7

Gandour, J. T., & Harshman, R. A. (1978). Crosslanguage Differences in Tone Perception: a Multidimensional Scaling Investigation. *Language and Speech*, *21*(1), 1–33. https://doi.org/10.1177/002383097802100101

Ho, C. S., & Bryant, P. (1997). Development of phonological awareness of Chinese children in Hong Kong. *Journal of Psycholinguistic Research*, *26*(1), 109–126. https://doi.org/10.1023/a:1025016322316

Law, S. P., Fung, R., & Kung, C. (2013). An ERP Study of Good Production vis-à-vis Poor Perception of Tones in Cantonese: Implications for Top-Down Speech Processing. *PLoS ONE*, *8*(1). https://doi.org/10.1371/journal.pone.0054396

Lee, C.-Y., Lekich, A., & Zhang, Y. (2014). Perception of pitch height in lexical and musical tones by English-speaking musicians and nonmusicians. *The Journal of the Acoustical Society of America*, *135*(3), 1607–1615. https://doi.org/10.1121/1.4864473

Lee, K. Y. S., Chan, K. T. Y., Lam, J. H. S., Van Hasselt, C. A., & Tong, M. C. F. (2015). Lexical tone perception in native speakers of Cantonese. *International Journal of Speech-Language Pathology*, *17*(1), 53–62. https://doi.org/10.3109/17549507.2014.898096

Li, M., & Dekeyser, R. (2017). Perception practice, production practice, and musical ability in L2 Mandarin tone-word learning. *Studies in Second Language Acquisition*, *39*(4), 593–620. https://doi.org/10.1017/S0272263116000358

Maggu, A. R., Lau, J. C. Y., Waye, M. M. Y., & Wong, P. C. M. (2021). Combination of absolute pitch and tone language experience enhances lexical tone perception. *Scientific Reports*, *11*(1), 1485. https://doi.org/10.1038/s41598-020-80260-x

Maggu, A. R., Wong, P. C. M., Antoniou, M., Bones, O., Liu, H., & Wong, F. C. K. (2018). Effects of combination of linguistic and musical pitch experience on subcortical pitch encoding. *Journal of Neurolinguistics*, *47*(March), 145–155. https://doi.org/10.1016/j.jneuroling.2018.05.003

Mok, P. P. K., & Zuo, D. (2012). The separation between music and speech: Evidence from the perception of Cantonese tones. *The Journal of the Acoustical Society of America*, *132*(4), 2711–2720. https://doi.org/10.1121/1.4747010

Ou, J., Law, S.-P., & Fung, R. (2015). Relationship between individual differences in speech processing and cognitive functions. *Psychonomic Bulletin & Review*, *22*(6), 1725–1732. https://doi.org/10.3758/s13423-015-0839-y

Ou, J., & Law, S. P. (2017). Cognitive basis of individual differences in speech perception, production and representations: The role of domain general attentional switching. *Attention, Perception, and Psychophysics*, *79*(3), 945–963. https://doi.org/10.3758/s13414-017-1283-z

Qin, Z., & Jongman, A. (2016). Does Second Language Experience Modulate Perception of Tones in a Third Language? *Language and Speech*, *59*(3), 318–338. https://doi.org/10.1177/0023830915590191

Shu, H., Peng, H., & McBride-Chang, C. (2008). Phonological awareness in young Chinese children. *Developmental Science*, *11*(1), 171–181. https://doi.org/10.1111/j.1467-7687.2007.00654.x

Tang, W., Xiong, W., Zhang, Y. xuan, Dong, Q., & Nan, Y. (2016). Musical experience facilitates lexical tone processing among Mandarin speakers: Behavioral and neural evidence. *Neuropsychologia*, *91*, 247–253. https://doi.org/10.1016/j.neuropsychologia.2016.08.003

Wong, P. C. M., & Perrachione, T. K. (2007). Learning pitch patterns in lexical identification by native English-speaking adults, *28*, 565–585. https://doi.org/10.1017/S0142716407070312

Wong, P. C. M., Skoe, E., Russo, N. M., Dees, T., & Kraus, N. (2007). Musical experience shapes human brainstem encoding of linguistic pitch patterns. *Nature Neuroscience*, *10*(4), 420–422. https://doi.org/10.1038/nn1872

Wu, H., Ma, X., Zhang, L., Liu, Y., Zhang, Y., & Shu, H. (2015). Musical experience modulates categorical perception of lexical tones in native Chinese speakers. *Frontiers in Psychology*, *6*(APR), 1–7. https://doi.org/10.3389/fpsyg.2015.00436

Yu, A. C. L. (2007). Understanding near mergers: The case of morphological tone in Cantonese. *Phonology*, *24*(1), 187–214. https://doi.org/10.1017/S0952675707001157

Zhao, T. C., & Kuhl, P. K. (2015). Effect of musical experience on learning lexical tone categories. *The Journal of the Acoustical Society of America*, *137*(3), 1452–1463. https://doi.org/10.1121/1.4913457

Zhu, J., Chen, X., & Yang, Y. (2021). Effects of Amateur Musical Experience on Categorical Perception of Lexical Tones by Native Chinese Adults: An ERP Study. *Frontiers in Psychology*. Retrieved from https://www.frontiersin.org/article/10.3389/fpsyg.2021.611189
