# Supplementary material for: Dissociation of tone merger and congenital amusia in Hong Kong Cantonese
Source: PLoS One. 2021 Jul 1;16(7):e0253982. doi: 10.1371/journal.pone.0253982 (PMC8248700; doi:10.1371/journal.pone.0253982)
Supplement: Attachment — Submitted filename: Response to Reviewers.docx [file pone.0253982.s003.docx]

# Response to Reviewers

Reviewer #2:

I think the authors have satisfactorily addressed my previous comments. The current manuscript is improved in various ways. Just some final minor comments:
***Response*:** Thank you very much for reviewing our manuscript and for the constructive feedback, which has helped us to strengthen the manuscript.

p.4: ‘Overall, three types of tone merger were reported: [+per][-pro] indicating good perception but poor production, [-per][+pro] indicating poor perception but good production, and [-per][-pro] where both perception and production are poor’ It is commendable that the authors have changed the terms they use for the three groups. I think ‘better’ and ‘poorer’ may be more appropriate than ‘good’ and ‘bad’.
***Response*:** The requested change has been made. Thanks.

p.18: ‘Any creaky voice or anomalies in the F0 values were excluded from analyses.’ They should report how much data was excluded. The lowest F0 in T4 is closely related to the creaky portion. More details should be given here.
***Response*:** More details including the percentage of F0 sampling points discarded from analysis due to creakiness have been added.

p.28: (J. Gandour, 1983; J. T. Gandour & Harshman, 1978; Z. Qin & Jongman, 2016)
p.31: (P. Loui, Alsop, & Schlaug, 2009; Psyche Loui, Guenther, Mathys, & Schlaug, 2008; Victoria J. Williamson, Liu, Peryer, Grierson, & Stewart, 2012)
They should remove the initials. Please check for citation consistence throughout the manuscript.
***Response*:** The author initials have been removed here and elsewhere in the manuscript as requested.

p.36: ‘One possible explanation is that the amusic participants recruited in the current study have rather severe impairments in musical pitch perception (see Figure 3) (Shao et al., 2016; Zhang & Shao, 2018).’ I think they need to point out explicitly how worse their participants were compared to those in the two previous studies to strengthen their argument.

***Response*:** The global scores of amusics in the current study and two previous studies have been added to support the argument.
